# Adding ursodeoxycholic acid to the endoscopic treatment and common bile duct stenting for large and multiple biliary stones: Will it improve the outcomes?

**DOI:** 10.1186/s12876-020-01523-5

**Published:** 2020-11-10

**Authors:** Ahmad Hormati, Mohammad Reza Ghadir, Seyed Saeed Sarkeshikian, Faezeh Alemi, Majid Moghaddam, Sajjad Ahmadpour, Abolfazl Mohammadbeigi, Gholam Reza Sivandzadeh

**Affiliations:** 1 Gastroenterology and Hepatology Diseases Research Center, Qom University of Medical Sciences, Qom, Iran; 2 Gastrointestinal and Liver Diseases Research Center, Iran University of Medical Sciences, Tehran, Iran; 3 Research Center for Environmental Pollutants, Qom University of Medical Sciences, Qom, Iran; 4 Department of Internal Medicine, Gatroenterohepatology Research Center, Shiraz University of Medical Sciences, Shiraz, Iran

**Keywords:** Cholelithiasis, Gallstones, Common bile duct, Ursodiol, Cholangiopancreatography, Endoscopic retrograde, Sphincterotomy, Endoscopic

## Abstract

**Background:**

The role of common bile duct (CBD) stenting in the establishment of bile stream in the elderly patients and the ones who are not good candidates for surgery due to not responding to treatments was well documented in previous studies. The current study aimed at investigating the effect of adding Ursodeoxycholic acid (UDCA) to CBD stenting alone in order to reduce the size of large and multiple CBD stones.

**Methods:**

Clinical outcomes including success rates in CBD stones clearance, incidence of pancreatitis, perforation, bleeding, as well as, decrease in size of stones and liver enzymes after a two-month period were assessed in the UDCA + CBD stenting group.

**Results:**

A total of 64 patients referring to Shahid Beheshti Hospital in Qom, Iran with multiple or large CBD stones (above three or larger than 15 mm) received standard endoscopic therapies and UDCA + CBD stenting (group B) and controls only received standard endoscopic therapies with only CBD stenting (group A). The mean reduction in the size of stones in group B was significantly higher than that of group A (3.22 ± 1.31 vs 4.09 ± 1.87 mm) (p = 0.034). There was no difference in the incidence rate of complications including pancreatitis, cholangitis, bleeding, and perforation between the two groups (P > 0.05).

**Conclusion:**

Adding UDCA to CBD stenting, due to decrease in the stone size and subsequently facilitation of the stones outlet, can be considered as the first-line treatment for patients with large and multiple CBD stones. Also, in the cases with large or multi stones may be effective in reducing size and subsequently stone retrieval.

*Trial registry* The study protocol was approved by the Ethics Committee of Qom University of Medical Sciences (ethical code: IR.MUQ.REC.1397.075); the study was also registered in the Iranian Registry of Clinical Trials (No. IRCT20161205031252N8). This study adheres to CONSORT guidelines.

## Background

Common bile duct (CBD) stone is found in approximately 7–12% of patients undergoing cholecystectomy for symptomatic gallstones, and is a common indication for endoscopic retrograde choangiopancreatography (ERCP). The CBD stones vary in size, from 1–2 mm to above 3 cm in diameter. ERCP with endoscopic sphincterotomy and stone retrieval with basket and balloon is the therapeutic options commonly used to treat CBD stones. It is estimated that roughly 85–95% of all CBD stones can be effectively treated with these common methods [1].

CBD stones with a maximum diameter of 1.5 cm can be removed with the endoscopic sphincterotomy technique. By increasing the size of stone, the success rate of these methods and the risk of complications such as perforation, cholangitis, and pancreatitis are increased; therefore, the utilization of methods with maximum success rates and minimum complications seems essential. Stones ≥ 15 mm should be broken up before extraction [2–4].

To treat stones called complicated stones, several methods including various lithotripsy techniques (electrohydraulic lithotripsy (EHL) and laser lithotripsy (LL), endoscopic papillary balloon dilatation (EPBD), sphincterotomy, and CBD stenting are applied. According to the results of different studies, EHL is associated with increased risk of duct rupture and EPBD with increased risk of pancreatitis. In older patients with concomitant critical illnesses that surgical procedures or other endoscopic measures may threaten their lives, the use of less invasive methods with minimal complications seems logic [5, 6].

Hydrophilic bile acids, such as ursodeoxycholic acid (UDCA), are used to treat CBD stones, especially cholesterol containing stones. Although the treatment of choice for cholelithiasis is cholecystectomy, patients with small cholesterol stones, without severe symptoms, and proper function of the gallbladder receive UDCA therapy, if they are not good candidates for surgery. Studies indicate that up to 60% of patients have a chance to completely recover from CBD stones with pharmacotherapies if they are good candidates for medical treatment [7, 8].

Regarding the high risk of complications in patients with large and multiple CBD stones, it is reasonable to adopt therapeutic methods with maximum efficacy in order to reduce the size of the stones. Several studies indicated the efficacy of CBD stenting following the endoscopic treatment to reduce the size of large CBD stones. The current study aimed at investigating the effect of adding UDCA accompanied by CBD stenting in order to reduce the size of stones and increase the success rate of endoscopic treatment in patients with large and multiple CBD stones [9].

## Methods

This randomized, controlled, clinical trial study was conducted on patients with multiple (≥ 3) and/or large (> 15 mm in diameter) CBD stones. The statistical sample was comprised of patients with large and multiple CBD stones that were candidates for endoscopic treatment by ERCP. The inclusion criteria were both ≥ 3 CBD stones and with ≥ 15 mm in diameter. The exclusion criteria were history of stomach, duodenum, or CBD surgery, having gastrointestinal stenosis, hemorrhagic disorders, high-risk cardiovascular diseases, and unwillingness to participate in the study.

The sample size required for the current study was determined 32 in each group using the sample size formula, based on the type 1 error as 0.05 and the type 2 error as 0.20.

The eligible patients after signing the informed consent form were randomly assigned to each of the therapeutic group A or B using the permuted block randomization method. Group A (control) underwent endoscopic therapy with ERCP and stent insertion, and the group B (intervention) received UDCA therapy in addition to ERCP and stenting, for two months. Treatments were appointed to groups A and B by coin tossing. In the current study, 10-Fr plastic stents, 8–14 cm length, were used for the subjects based on their CBD length. UDCA tablets 300 mg (Tehran Pharm Company) were administered to the subjects three times daily per os.

The information of patients in both groups were recorded in a checklist including demographic characteristics such as age, gender, height, and weight, history of underlying diseases, history of pancreatitis, and the size and number of CBD stones (measured before ERCP). The liver transaminases, alkaline phosphatase, and serum bilirubin levels were measured before the procedure and recorded in the patient's checklist. Patients then underwent ERCP. ERCPs were conducted by three expert gastroenterologists. The number and size of the stones were recorded by fluoroscopy during the ERCP, after the injection of contrast material and radiography. The CBD stent was then inserted. Information on complications during the procedure including perforation of the CBD and bleeding was recorded in the patient's checklist.

Patients in group B were treated with UDCA and both groups underwent ERCP again after two months in order to remove the stent. In the second turn of the ERCP, complications were monitored during the procedure and after 24 h. The success rate, defined as a reduction in the number and/or size of CBD stones, was evaluated through ERCP and the results were recorded in the checklist.

Data were analyzed with SPSS 18 software using chi-square, independent and paired samples t-test, and analysis of covariance (ANCOVA). Mean and standard deviation were used to express the data. The P-value < 0.05 was considered as the level of significance.

## Results

Out of 64 patients enrolled in the current clinical trial, thirty-two cases in the control group underwent CBD stenting alone (group A) and the other 32 cases in the intervention group underwent CBD stenting + UDCA (group B). There were 16 females in each group and there was no difference in gender distribution between the groups. The mean age of patients in groups A and B was 44.31 ± 12.55 and 41.33 ± 41.3 years, respectively. The demographic variables including age and BMI were not significantly different between the two groups. Before ERCP, the average number of stones in groups A and B were 3.34 ± 2.06 and 3.56 ± 2.26 respectively, that were similar in the two groups (P = 0.67). The findings are shown in Table 1. The indication for ERCP in all patients was biliary obstruction due to stone and no evidence of malignancy was detected in cases. Mean levels of liver transaminases, bilirubin, and alkaline phosphatase summarized in Table 2 showed no significant differences between the two groups.

**Table 1:** Findings and complications in both groups underwent to only CBD stenting (group A) and UDCA + CBD stenting (group B)

Findings and complications		Control or group A N (%)	Intervention or Group B N (%)	P-value*
Pancreatitis	No	29 (90.6)	27 (87.1)	0.880
	Mild	2 (6.2)	3 (9.7)	
	Moderate	1 (3.1)	1 (3.1)	
	Severe	0	0	
Cholangitis	No	31 (96.9)	31 (96.9)	1
	Yes	1 (3.1)	1 (3.1)	
Perforation	No	32 (100)	32 (100)	1
	Yes	0	0	
Sphincterotomy	No	32 (100)	32 (100)	1
	Yes	0	0	
Stone removal	Yes	27 (84.4)	30 (93.8)	0.230
	No	5 (15.6)	2 (6.2)	
Diverticula	Yes	28 (87.5)	26 (81.2)	0.491
	No	4 (12.5)	6 (18.8)	
PD stent	Yes	2 (6.2)	3 (9.4)	0.641
	No	30 (93.8)	29 (90.6)	
PD Cannulation	Yes	25 (78.1)	30 (93.8)	0.154
	No	5 (15.6)	2. (6.2)	
GIB	Yes	5 (16.6)	3 (9.4)	0.450
	No	27 (84.4)	29 (90.6)	

*Based on Chi-square test

**Table 2:** Comparison of liver tests and reduction in the size and number of stone between two groups underwent to only CBD stenting (group A) and UDCA + CBD stenting (group B)

Variable	Control (Group A)	Intervention (Group B)	P-value*
Stone size in mm Mean (SD)	3.22 ± 1.31	4.09 ± 1.87	0.034
Number of stones Mean (SD)	3.34 ± 2.06	3.56 ± 2.26	0.67
Test, Unit#	Control (Group A)	Intervention (Group B)	P-value*
SGPT (ALT), U/L	184.38 ± 134.44	196.34 ± 114.5	0.754
SGOT (AST), U/L	141.31 ± 99.72	153.97 ± 114.5	0.639
Total Bilirubin, mg/dL	5.78 ± 1.64	5.47 ± 1.98	0.495
Direct Bilirubin, mg/dL	4.13 ± 1.18	4.06 ± 1.64	0.862
Amylase Serum, U/L	120.8 ± 172.29	127.19 ± 167.3	0.881

*ALT* Alanine aminotransferase; *AST* Aspartate aminotransferase

*Based on Independent T-test

^#^Reference range for ALT, AST, total Bilirubin and direct bilirubin are 13–40 U/L, 11–37 U/L, 0.1–1.2 mg/dL and 0–0.3 mg/ dL respectively

CBD cannulation was successfully performed in 86% of the patients and the success rate in the two groups had no difference (P = 0.154). The rate of complications during and after ERCP, including pancreatitis, cholangitis, and bleeding, summarized in Table 1, showed no significant differences between the two groups. ERCP-related perforation was not observed in any of the groups.

The average size of stones after two months in groups A and B were 3.22 ± 1.31 and 4.09 ± 1.87 mm, respectively that were significantly higher in group B (P = 0.034). The success rate of CBD stones clearance was 27 (84.4%) in group A and 30 (93.8%) in group B. There was no significant difference between two groups in terms of stone clearance rate (P = 0.230).

## Discussion

CBD stones vary in size, and most of them are treated with standard procedures. However, in less than 50% of cases, endoscopic sphincterotomy is difficult because of abnormal anatomy of the duct, abnormal stone location, large size, and high number of stones are effective in this condition [1, 2, 10–13]. Several methods are proposed for the treatment of large stones of which endoscopic CBD stenting is considered as an effective alternative [1, 5]. Reduction in the size and number of CBD stones is reported after two months of inserting the stent with a high success rate [1, 5, 9].

A number of studies showed the efficacy of pharmacotherapy in the treatment of CBD stones with hydrophilic bile acids, especially UDCA. It is also used in the treatment of chronic cholestatic diseases, such as primary biliary cirrhosis (PBC), as a useful and effective therapeutic option to protect the liver and slowdown the progression of liver damage. UDCA inhibits biliary cholesterol secretion, decreases intestinal absorption of cholesterol, increases hepatocyte bile secretion, and improves the evacuation of gallbladder of bile and other constituents. It also improves the contraction of gallbladder muscle and reduces inflammation in its wall. However, gallstones are treated medically with UDCA in cases with the mild clinical symptoms, stones smaller than 5–10 mm in diameter, and appropriate function of the gallbladder. The presence of calcium salts in the gallstones, observed as calcification in CT images, reduces the efficacy of UCDA therapy [14–17]. However, clinical studies show that if good candidates for medical treatment are selected, up to 60% are completely cleared of CBD stones after 12–24 months of treatment [7, 8].

The current study aimed at examining the efficacy of the combination of two methods of stenting and UDCA therapy in reducing the size of large and number of multiple CBD stones. The results showed that the utilized method could reduce the size of stones by 4 mm in average that was significantly higher than that of the control group. Also, the number of CBD stones removed from CBD in the intervention group was higher than the control group (93.8% vs. 84.4%); however, the difference was statistically insignificant (Table 1).

Pancreatitis is one of the most important diseases that predispose ERCP to complications. The overall incidence of pancreatitis was 11% in the present study, with three cases in the control group and four in the intervention group. There was no significant difference in the incidence of pancreatitis between the two groups. Pancreatitis is identified as the most common post-ERCP complication, and its prevalence generally varies from 3 to 5% in studies, but also can vary from 1 to 16% depending on selected patients [14–16]. In the present study, considering the patients conditions with large and multiple stones, procedures were generally more risky and the high rate of pancreatitis was expected in comparison with the total reported rate.

Cholangitis was observed in two patients (3.13%), of which one was in the control group and the other in the intervention group. The incidence rate of cholangitis was also different in studies; it was 3.8% in the study by Hong [17] that was similar to that of the current study, but in the study by Horiuchi [18] it was 13%; the different results can be attributed to patients’ differences in various studies.

In the recent study, the success rate in complete clearance of CBD stones was 93.8% in the intervention group and 84.4% in the control group. The results was consistent with those of previous studies, including Horiuchi (93%) [18], Hong (94%) [17], Hui (94.7%) [19], Fan (95.6%) [4] and Ye (94.1%) [6].

Compared to our previous studies, the additional effects of this study is associated to this fact that the prescribing UDCA along with CBD stenting leads to a further reduction in the size of the stones without increasing complications [9, 20]. However, according to the findings, there was no significant difference in CBD stones clearance rate. It should be noted that as a rule, UDCA is used in long-term treatments (at least 12–24 months), and such circumstances lead to a significant reduction in the size of the stones, especially cholesterol stones. The main limitation of this study is related to the duration of pharmacotherapy in the current study which was much shorter than its routine duration (2 months vs. 12 months). Also, there was also no screening for stones in terms of the type and presence of calcification, especially peripheral calcifications. Given to the fact that UDCA is less effective in cases of calcified stones and patients characteristics is very important in response to UDCA therapy, subsequent clinical trials with larger populations and a more precise selection of patients can be useful in obtaining more conclusive results about the efficacy of the utilized method in the clearance of difficult CBD stones.

## Conclusion

Generally, it can be concluded that while common bile duct (CBD) stenting is an effective method for retaining the continuity of the bile flow in the elderly patients and the ones who are not good candidates for surgical treatment, adding UDCA to this treatment can improve the outcomes. Also, in patients with large and multiple stones, procedures were generally accompanied with more risk and may lead to higher rate of pancreatitis. Results of the current study demonstrated that combination of ERCP and CBD stenting with medical treatment with UDCA, is more effective in reducing of the size of CBD stones than CBD stenting alone. Also, we achieved to high success rate in complete clearance of CBD stones. Beside of these benefits we also do not find any significant differences in the incidence of pancreatitis between the two groups. Therefore, we conclude that this new combination method provided us further reduction in the size of the stones in patients with large and multiple biliary stones without increasing complications that were not reported previously.

## Data Availability

All data and materials are available from the corresponding author.

## Abbreviations

CBD: Common bile duct
UDCA: Ursodeoxycholic acid
ERCP: Endoscopic retrograde choangiopancreatography
EHL: Electrohydraulic lithotripsy
LL: Laser lithotripsy
EPBD: Endoscopic papillary balloon dilatation
PBC: Primary biliary cirrhosis
